# Use of carefully titrated ultra‐low doses of alteplase in infected haemothorax caused by initial alteplase use to drain loculated malignant pleural effusion

**DOI:** 10.1002/rcr2.1202

**Published:** 2023-08-09

**Authors:** Jeong Suk Oh, Chris Yoo, Donny Wong

**Affiliations:** ^1^ Department of Respiratory Medicine North Shore hospital Auckland New Zealand

**Keywords:** bleeding, intrapleural fibrinolytics, loculations, pleural effusion

## Abstract

Alteplase as a fibrinolytic can be used to break up fibrin to encourage clot breakdown for clinical use. In the pleural space, it is used for symptomatic loculated malignant pleural effusions and pleural infections and can potentially avoid the need for surgical intervention. The optimal dose and dosing regimen of intrapleural fibrinolytics is still unknown. Although generally considered safe, bleeding is a serious potential complication and studies are ongoing to try and determine the lowest effective dose of alteplase to successfully treat pleural infections. This case highlighted the safe use of very low doses of alteplase ranging from 0.25 to 0.5 mg following pleural bleeding after the use of alteplase to treat a patient with symptomatic malignant loculated effusion. It demonstrates once pleural bleeding has stopped, there is a role for carefully titrated intrapleural alteplase use to avoid surgery.

## Graphical abstract

This case highlights the safe use of very low doses of alteplase ranging from 0.25 to 0.5 mg following pleural bleeding after the use of alteplase to treat a patient with symptomatic malignant loculated effusion and may demonstrate there may be a use for carefully titrated alteplase in the pleural space after pleural bleeding has stopped to treat pleural infection.

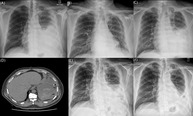

## INTRODUCTION

Studies have shown that pleural infections could be successfully managed using intercostal chest drains and intrapleural alteplase and dornase.^1^ There has been ongoing interest in using lower doses of intrapleural fibrinolytics to treat pleural infections given the risks of bleeding, although uncommon, can be serious.^2^ The optimal dose and dosing regimen of intrapleural fibrinolytics are still unknown.

We wish to highlight the medical management of pleural infections with very low dose alteplase, guided by previous literature,^1^, ^2^, ^3^, ^4^ when clinicians face dilemmas with bleeding associated with intrapleural fibrinolytics.

## CASE REPORT

A 68‐year‐old Samoan man with a KRAS G13C positive stage IV lung adenocarcinoma undergoing chemotherapy had an indwelling pleural catheter (IPC) inserted for symptomatic management of his malignant pleural effusion in the context of a trapped lung. He had been on aspirin as well.

**FIGURE 1:**
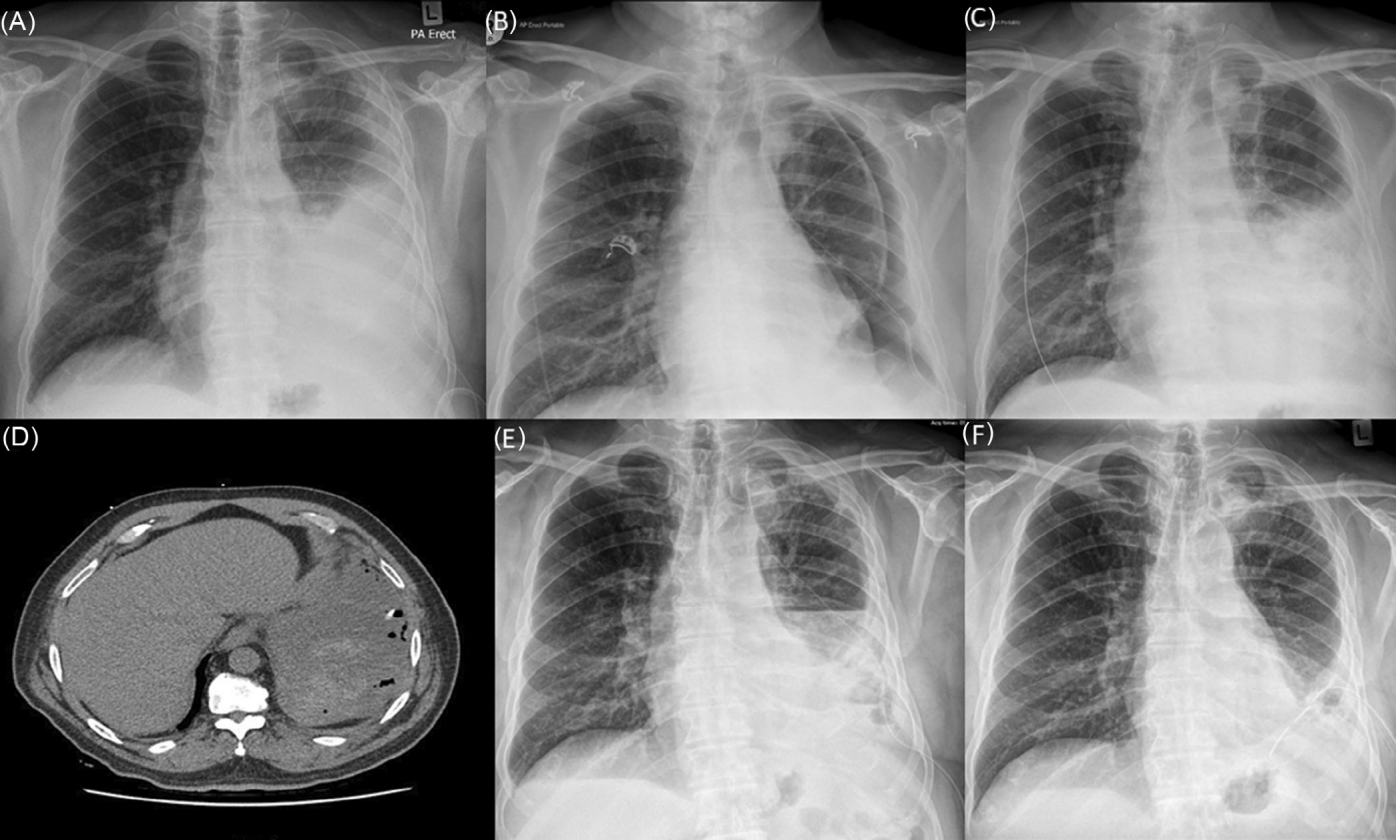
Chest radiograph and computed tomography (CT) images during this patient's treatment course. (A) Pre‐intrapleural fibrinolytics: left pleural effusion; (B) after initial 5 mg intrapleural alteplase with resolution of pleural effusion and evidence of trapped lung; (C) re‐accumulation of pleural fluid 24 hours after 5 mg intrapleural alteplase; (D) CT images showing hyperdense material in the posterior and basal part of the pleural effusion suggestive of haemorrhage; (E) after 1 mg of intrapleural alteplase; (F) chest radiograph after a single 0.25 mg and two 0.5 mg alteplase doses were given with improvement in the left pleural effusion.

One month after his IPC insertion, he reported worsening dyspnoea with poor drainages in the community. A chest x‐ray showed a large pleural effusion (Figure 1).

He was admitted to the hospital for troubleshooting where the IPC flushed well with saline however pleural fluid was unable to be aspirated. This suggested the pleural effusion was loculated and 5 mg of intrapleural alteplase was given. All alteplase doses were given alongside 5 mg dornase in this case report.

Over the next 24 h, two litres of haemoserous fluid was drained. His dyspnoea improved and the pleural effusion size decreased significantly on his chest radiograph (Figure 1). The serum and pleural fluid haematocrit levels were 0.36 and 0.056, respectively.

Over the following 48 h, he became pyrexial and his C‐reactive protein rose from 96 mg/L to a peak of 295 mg/L during the admission. His serum haemoglobin count also decreased from 125 to 95 g/L. A repeat chest radiograph showed the pleural effusion re‐accumulated (Figure 1). He was started on intravenous ceftriaxone and metronidazole. The serum activated partial thromboplastin time was 44 s and prothrombin ratio was 1.3. He was not thrombocytopenic. A CT thoracic angiogram was undertaken which showed a hydropneumothorax with multiple loculations and hyperdense material suggestive of recent pleural haemorrhage (Figure 1).

We deemed clinically that he had pleural bleeding given the sequence of chest x‐rays and CT appearance combined with the serum haemoglobin drop. After a 24 h waiting period, a lower second dose of 1 mg alteplase was instilled. However, further pleural bleeding was confirmed following the 1 mg alteplase (Figure 1), as analysis of serum and pleural fluid haematocrit levels post 1 mg dose showed 0.30 and 0.161, respectively. Other pleural fluid analysis showed a pH of 7.1 and it became neutrophilic predominant with culture negative and cytology showing only blood. The IPC was not swinging and there were concerns the IPC was not optimally placed thus a radiological guided 14F chest drain was inserted to the anterior pocket.

Over the next 72 h he remained pyrexial with elevation of his inflammatory markers and poor drainage. The surgical option of a mini‐thoracotomy and open pleural drainage was considered. We opted for the non‐surgical approach given his co‐morbidities. The IPC was removed to exclude the catheter as a source of infection. A large bore chest drain was inserted after a dose of 0.25 mg alteplase was given the same day resulting in an output of 525 mL with a lighter haemoserous content. His haemoglobin count was stable.

The next day, bedside ultrasound showed areas of persisting echogenic fluid in pockets. The intrapleural alteplase dose was increased to 0.5 mg in an attempt to break down the loculated effusion further inferiorly in the pleural space. A further 500 mL came out of the chest drains over the next 24 h. There was no evidence of pleural bleeding clinically, based on his pleural aspirates and on serial haemoglobin counts. A second 0.5 mg intrapleural alteplase was instilled on the third day with two litres of amber coloured fluid coming out. There was a reduction in his pleural effusion on his chest radiograph (Figure 1) alongside improvement in his inflammatory markers.

His chest drains were removed and was discharged with 4 weeks of outpatient intravenous antibiotics. He died 10 months later from progression of his cancer with no further pleural interventions.

## DISCUSSION

The doses of alteplase and dornase used in the original MIST‐2 trial were 10 mg and 5 mg, respectively.^1^ The risk of pleural bleeding prompted clinicians to trial lower doses of fibrinolytics to treat pleural infections.^2^ A case report also highlighted successful treatment of a case of pleural infection with 1 mg alteplase in a patient with coagulopathy.^4^ The ADAPT‐2 study is the second series of studies lowering the initial dose of alteplase after a previous study looked at 5 mg, reducing the dose to 2.5 mg‐ it observed an 88.4% treatment success rate without requiring cardiothoracic surgery.^2^

In our case, there is proof of concept that despite the initial administration of alteplase causing pleural bleeding, there may well be for each patient an individual therapeutic window for alteplase use in the pleural space. In this case much lower doses of alteplase could be used after pleural bleeding had stopped to safely treat pleural infection. It was arbitrary how the lower doses were selected in this case and it was guided by ease of drawing up alteplase from 10 mg ampoules.

Further studies investigating even lower doses of alteplase than what was reported in the ADAPT‐2 study are underway^2^ which would provide further data into the lowest and safest effective dose of alteplase use in the pleural space.

## AUTHOR CONTRIBUTIONS

Jeong Suk Oh was involved in the analysis and interpretation of data for the work and manuscript write up, Chris Yoo was involved in the analysis and interpretation of data for the work and Donny Wong was involved in the design of the work, revising for important intellectual content and reviewing the manuscript for publication.

## CONFLICT OF INTEREST STATEMENT

None declared.

## ETHICS STATEMENT

The authors declare that appropriate written informed consent was obtained for the publication of this manuscript and accompanying images.

## Data Availability

The data that support the findings of this study are available on request from the corresponding author. The data are not publicly available due to privacy or ethical restrictions.
